# Arylimidamides Have Potential for Chemoprophylaxis against Blood-Transmitted Chagas Disease

**DOI:** 10.3390/pathogens12050701

**Published:** 2023-05-12

**Authors:** Bruno Lisboa Timm, Aline Nefertiti Silva da Gama, Marcos Meuser Batista, Denise da Gama Jaén Batista, David W. Boykin, Harry P. De Koning, Maria de Nazaré Correia Soeiro

**Affiliations:** 1 Laboratório de Biologia Celular, Instituto Oswaldo Cruz, Fundação Oswaldo Cruz, Rio de Janeiro 21045-900, Brazil; timmbruno@gmail.com (B.L.T.);; 2 Department of Chemistry, Georgia State University, Atlanta, GA 30303, USA; 3 School of Infection and Immunity, College of Medical, Veterinary and Life Sciences, University of Glasgow, Glasgow G43 2DX, UK

**Keywords:** *Trypanosoma cruzi*, blood bank, arylimidamides, chemoprophylaxis, Chagas disease

## Abstract

Chagas disease (CD) affects over 6 million people worldwide and can be transmitted iatrogenically. Crystal violet (CV) was previously used for pathogen reduction but has harmful side-effects. In the present study, three arylimidamides (AIAs) and CV were used to sterilize mice blood samples experimentally contaminated with bloodstream trypomastigotes (BT) of *Trypanosoma cruzi*, at non hemolytic doses. All AIAs were not toxic to mouse blood cells until the highest tested concentration (96 µM). The previous treatment of BT with the AIAs impaired the infection establishment of cardiac cell cultures. In vivo assays showed that pre-incubation of mouse blood samples with the AIAs and CV (96 µM) significantly suppressed the parasitemia peak, but only the AIA DB1831 gave ≥90% animal survival, while vehicle treated samples reached 0%. Our findings support further studies regarding the potential use of AIAs for blood bank purposes.

## 1. Introduction

*Trypanosoma cruzi* is a vector-borne protozoan pathogen that causes Chagas disease (CD), which is responsible for high morbidity and mortality rates in endemic areas of Latin America. This neglected tropical disease affects more than 6 million people worldwide, mainly in resource-poor rural areas where public health services face substantial constraints [1,2]. In addition to Central and South America, CD also represents a public health concern in non-endemic areas such as North America, Asia, Oceania and Europe; this is mainly attributed to human migration and the infection often being asymptomatic, i.e., unnoticed [3,4,5,6,7]. CD has two sequential clinical stages: the acute phase, defined by a short, oligosymptomatic state with patent parasitemia, followed by a long chronic phase characterized by subpatent and apparently intermittent parasitemia. Approximately 30% of the infected people develop progressive cardiac and digestive abnormalities over the years [8,9,10]. The available chemotherapy, based on two old nitro-derivatives, benznidazole and nifurtimox, is not satisfactory, especially during the later chronic phase of the disease [11,12,13,14]. Both require a long period of drug administration and induce several side effects such as fatigue, nausea, vomiting, anorexia, central nervous system changes, headache, dizziness or vertigo, as well as mood swings, resulting in withdrawal rates around 20% [15,16,17]. Despite CD being the main cause of mortality in Latin America due to parasitic infection, fewer than 10% of the infected people are diagnosed and less than 1% are treated [18].

*T. cruzi* can be transmitted through different mechanisms including the vectorial route (through infected triatominae feces), blood transfusion, organ transplantation, laboratory accidents, congenital transmission, and orally, due to contaminated food [19,20,21,22]. Several outbreaks of orally transmitted CD have been reported and this route currently represents the major cause of new cases in several countries including Brazil; this is mostly attributable to a lack of good handling practices in food processing [21,22,23,24].

Due to previous programs such as the Southern Cone and the Andean Countries Initiatives, the transmission of *T. cruzi* through vector (domiciliary infestation by *Triatoma infestans*), blood bank and organ transplantation were significantly reduced in Latin America [25,26]. However, in recent decades, the decentralization of public health services, linked to increased poverty, and the lack of infrastructure and resources, have enabled the reemergence of CD, which represents a real threat to population health, especially in hotspot areas such as the Gran Chaco (Bolivia and Argentina) [27,28]. Additionally, the persistent and/or recurrent house infestations with triatomines, even after chemical spraying, are interconnected with domestic and peridomestic infection cycles, supporting control strategies that target both habitats [1]. Moreover, the diagnosis and treatment of infected women of reproductive age, and the diagnosis of pregnant infected women and infected babies, must be implemented to mitigate vertical transmission and some projects are underway such as Cuida Chagas and Integra Chagas [29].

Iatrogenic transmission, mainly related to blood transfusions, was first reported by Salvador Mazza (1936) [30] in Argentina and later confirmed by Pedreira de Freitas and coworkers in Brazil (1952) [31]. Infected blood poses a significant problem in highly endemic cities such as Santa Cruz de la Sierra and Cochabamba in Bolivia, where up to one-half of blood donors can be infected with *T. cruzi* [4]. Nowadays, the risk of iatrogenic transmission varies according to the CD endemicity of the region. A prevalence of up to 3% has been reported in blood banks in endemic regions [24], reinforcing the need for a sustainable universal screening in all blood banks, with reliable and reproducible high performance serological tests (99–100%) validated via confirmatory assays (≥95% of specificity). Additionally, some non-endemic regions present similar or higher prevalence of *T. cruzi* in blood bank stocks than endemic areas, possibly due to the high number of imigrants, showing that control protocols must be expanded to further geographical areas [24].

Nussenszweig and coworkers were the first to report the effect of gentian violet (GV), a mixture of three dyes (crystal violet, methyl violet, and brilliant green), on trypomastigotes of *T. cruzi*, proposing its use in blood banks to prevent transfusional CD [32]. This triarylmethane cationic dye began to be employed in blood banks in Latin America in the 1960s, as recommended by the WHO [33,34]; however, one of the main concerns about its use was due to its purple color staining the skin and mucosae of the recipients [35,36,37].

Given the large number of asymptomatic, undiagnosed, and untreated infected people, iatrogenic transmission is still one of the main transmission routes for *T. cruzi*, even in Latin America, justifying the search for new chemoprophylactic agents.

Amidine-containing compounds such as pentamidine are DNA minor groove binders with a broad spectrum of activities against human and veterinary pathogens, especially protozoa [38,39,40]. Due to their promising microbicidal activity but rather poor bioavailability and high toxicity, many analogues, and derivatives, including pro-drugs, have been synthesized and screened in vitro and in vivo to improve their selectivity and pharmacological properties [41]. A class of amidine derivatives, initially described as ‘reversed amidines’ but now referred to as arylimidamides (AIAs), demonstrated high potency against several parasites, particularly *T. cruzi*, *Leishmania* sp., *Neospora caninum*, *Besnoitia besnoiti* and *Echinococcus multilocularis* [42,43,44,45].

Some AIAs such as DB745, DB766 and DB1831 maintain a high trypanocidal activity at 4 °C in the presence of 96% mouse blood, being at least 30-fold more effective than crystal violet [46,47,48]. In the present study, the potential application of these three AIAs in the chemoprophylaxis of *T. cruzi* infection was investigated through in vitro and in vivo assays.

## 2. Materials and Methods

### 2.1. Compounds

**Figure 1:**
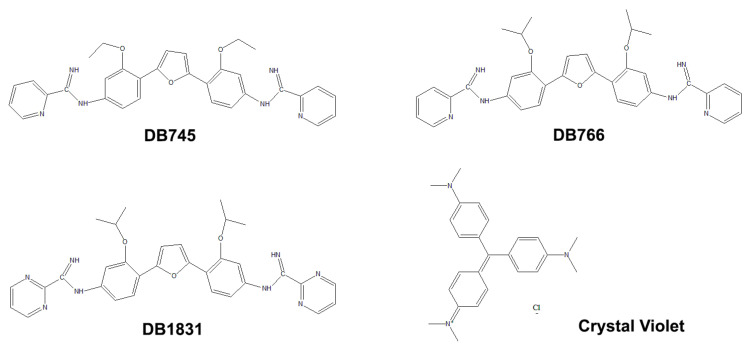
Structural formulae of the compounds.

The synthesis of the AIAs (DB745, DB766 and DB1831) (Figure 1) was performed as reported [49,50]. Stock solutions of the compounds were prepared in dimethyl sulfoxide (DMSO); the final DMSO concentration never exceeded 0.6% in assays, which did not exert any toxic effect on the parasite or the mammalian host cells. Stock solutions of crystal violet (CV, Figure 1) were prepared in deionized water and stored at 4 °C. 

### 2.2. Hemolytic Assay

The blood of non-infected Swiss Webster mice (18–20 g) was collected and diluted in phosphate-buffered saline (PBS) to a concentration of 25% of blood cells. In 96-well microplates, 100 µL of the mouse blood suspension was added to the same volume of DB745 or DB766 at concentrations ranging from 2.4 to 192 µM. After incubation at 4 °C for 24 h, the samples were centrifuged (5 min/540× *g*) and a 100 µL aliquot was transferred to another microplate and the levels of hemoglobin measured at 540 nm. Deionized water was used as control for 100% hemolytic activity, and PBS as a negative control. The degree of hemolysis was calculated by the variation index (VI) that corresponds to the ratio of optical density (OD) of the treated sample/positive control [47]. The reading of the OD of the samples was performed in the SpectraMax^®^ Plus 384 Absorbance Plate Reader spectrophotometer (540 nm) and duplicate samples were run in at least two assays.

### 2.3. Parasites

Bloodstream trypomastigote (BT) forms of the Y strain were obtained from infected male Swiss mice, at peak parasitemia. The purified parasites were resuspended in Dulbecco’s modified Eagle’s medium (DME) supplemented with 10% FBS (DMES) [48,51].

### 2.4. In Vitro Infectivity Profile of Bloodstream Trypomastigotes Treated with AIAs

To verify the infectivity profile of BT exposed to the AIAs before their interaction with mammalian host cells, mouse blood infected with 5 × 10^4^ trypomastigotes/mL was incubated for 2 h with the EC_50_ value of each compound (6.4 µM for DB745, 1.14 µM for DB766, respectively [45,52]. Then, the parasite suspensions were centrifuged (3000 rpm/10 min) and the pellet resuspended in RPMI, quantified through light microscopy [53], and used for the infection of Swiss embryo mouse cardiac cell cultures (CC) seeded (15 × 10^4^ cells/well) in 24-well plates (10:1 parasite–cell ratio). After 24 h of parasite–host cell interaction, the infected cultures were washed to remove free parasites and maintained at 37 °C in an atmosphere of 5% CO_2_ and air and the culture medium replaced every 24 h. Afterwards, untreated and treated infected cardiac cells (CCs) were fixed and stained with Giemsa and examined via light microscopy [47,52]. Only characteristic *T. cruzi* nuclei and kinetoplasts were counted as surviving parasites since irregular structures could mean parasites undergoing death. The activity of the AIAs was estimated by calculating the infection index (II), which corresponds to the percentage of infected cells multiplied by the average number of intracellular parasites per infected host cell [54]. Duplicate assays were run in the same plate and at least two independent assays were performed as standard.

### 2.5. In Vivo Infectivity Profile of Bloodstream Trypomastigotes Treated with AIAs

Male and female Swiss Webster mice (18–20 g) were obtained from the Fundação Oswaldo Cruz animal facilities (ICTB, Rio de Janeiro, Brazil). Mice were housed at a maximum of five per cage, kept in a specific pathogen-free (SPF) room at 20 to 24 °C under a 12-h/12 h light/dark cycle and provided sterilized water and chow ad libitum. The animals were allowed to acclimate for 7 days before the start of the experiments.

For evaluation of the chemoprophylactic potential of the AIAs and CV in vivo, the mice were inoculated (via i.p.) with 10^4^ trypomastigotes into 200 µL of: (a) infected untreated blood samples; (b) infected using blood samples pre-treated for 2 h with the compounds at a concentration corresponding either to their EC_50_ or with 96 µM of one of the AIAs. 

### 2.6. Parasitemia and Mortality Rates

Parasitemia of individual mice was daily checked (until 10 dpi, and then, twice a week) by direct microscopic counting of parasites in 5 µL of blood taken via tail prick [45,55]. Body weight was evaluated weekly, and mortality was checked daily until 40 days post-treatment and expressed as the percentage of survival [56]. Thirty days after treatment the animals were immunosuppressed with cyclophosphamide (50 mg/kg) in three cycles of 4 consecutive days/week and the parasitemia and mortality rates were evaluated for 4 weeks [57].

### 2.7. Ethical Approval

All procedures were carried out in accordance with the guidelines established by the FIOCRUZ Committee of Ethics for the Use of Animals (CEUA LW-16/2013, CEUA L038-2017).

### 2.8. Statistical Analysis

The analyses were obtained through nonlinear regression analysis carried out using GraphPad Prism v 9.0 (GraphPad Software, San Diego, CA, USA). Statistical analysis was performed using an ANOVA single-factor test with the level of significance set at *p* ≤ 0.05.

## 3. Results

### 3.1. Hemolytic Action of Arylimidamides DB745 and DB766

We aimed to reproduce the conditions of a prophylactic protocol for CD, and three AIAs and CV were selected, based on previously published data that demonstrated their sustainable activity against BT forms under the storage conditions of blood banks (incubation of parasites in 96% of mouse blood at 4 °C).

To investigate any potential toxicity on blood cells, the samples were obtained from mice and incubated with DB745 and DB766. Our data revealed that the compounds, at concentrations of up to 96 µM, did not induce hemolysis, while our positive control, performed using sterilized water, did induce erythrocyte lysis (Table 1). The hemolytic potential of CV was not evaluated since its characteristic color would interfere with the colorimetric readings and since it has been used safely in the past at blood banks to prevent transfusional CD.

### 3.2. In Vitro Infectivity Profile of Bloodstream Trypomastigotes Treated with AIAs

We next investigated whether pre-exposure of T. cruzi to AIAs diminished their cellular infectivity and proliferation in cardiac cells. The CC were exposed to trypomastigotes (1:10 ratio) that had been pretreated with AIAs at EC_50_ concentration, or vehicle.

As depicted in Table 2, the untreated parasites infected 23 and 26% of the infected host cells, with 1.6 and 18 parasites per infected cells, and infection indexes of 38 and 466 after 24 and 72 h of parasite invasion, respectively. When BT were first incubated with the AIAs for 2 h, statistically significant (*p* < 0.05) reductions of 44 and >99% in the percentage of infected CC were noticed after 24 and 72 h of parasite–host cell interaction. At both time points DB766 was highly significantly more effective than DB745. Most importantly, while the percentage of infected cells doubled between 24 and 72 h after treatment with DB745, infectivity fell from 1% to 0.1% with DB766, showing that parasite viability was fatally affected by the drug and that the parasite still died, even after successful infection, leaving the cardiac cells apparently uninfected. Regarding the number of parasites per infected host cell, while the pre-incubation of BT with DB745 only slightly impaired the parasite multiplication, DB766 greatly reduced the total parasite load, with decreases ranging from 69 to 98% after 24 and 72 h of CC infection and resulting in a >99% reduction in the II (Table 2).

Since in vitro results revealed (i) the low hemolytic potential of up to 96 µM of both AIAs and (ii) that a subpopulation of motile/live parasites after pre-treatment with DB745 displayed a greatly reduced ability to invade CC, which in the case of DB766 was almost abolished, in vivo assays were next performed. Additionally, the AIA DB1831 was included in the further in vivo analysis due to its previously reported prophylactic characteristic [47].

### 3.3. In Vivo Infectivity Profile of Bloodstream Trypomastigotes Treated with AIAs

The in vivo assays were conducted by inoculating experimental mice with artificially infected mouse blood samples that were first treated for 2 h with DB745, DB766, or DB1831, each at their EC_50_ concentration and at 96 µM, which are non-toxic concentrations for mammalian blood cells. In addition, the AIA DB1831 was included at this point, due to it also displaying high potential for blood bank chemoprophylaxis [47]. The first set of assays was performed using male Swiss mice inoculated with bloodstream trypomastigotes treated with the compounds immediately before the infection. The finding demonstrated that when the EC_50_ values were used to treat the inoculated mouse, except for one single assay using DB745 that reached a reduction of >98%, the pre-treatment with the three AIAs resulted in a 39–46% lower parasitemia peak. The parasitemia data revealed that none of the compounds were able to prevent the parasitemia after pre-treatment at EC_50_ (Table 3).

The AIAs, at their EC_50_ values, did not protect against animal mortality induced in this acute infection model. However, when the blood samples were pre-incubated with 96 µM of each drug, a statistically significant and very large suppression (*p* < 0.05) of the parasitemia peak was achieved (87–100%) for all AIAs and for the CV control (Table 3). Although complete clearance of parasitemia was observed in 60% of the infected mice treated with 96 µM DB1831, even after immune suppression with cyclophosphamide, a very low but positive parasitemia was detected in all of the experimental groups, including the CV-treated mice, as evaluated using the Pizzi method (Table 3).

#### In Vivo Infectivity Profile Bloodstream Trypomastigotes Treated with AIAs in Female and Male Models

**Figure 2:**
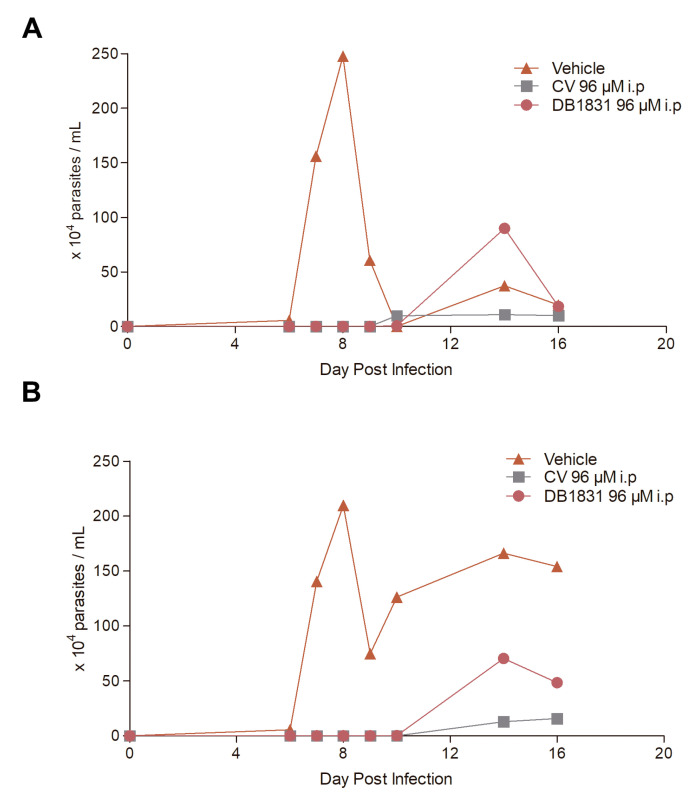
In vivo effect of the pre-treatment of bloodstream trypomastigote inoculation for 2 h with 96 μM of DB1831 and CV. Parasitemia in mice given vehicle-treated inoculum is also shown. The parasitemia curve is presented in the experimentally infected in female (**A**) and male (**B**).

Since, among the evaluated AIAs, only DB1831 at 96 µM led to full clearance of the parasitemia, the subsequent experiment was performed with only this condition, using CV 96 µM as the standard control, verifying the potential susceptibility profile in male and female animals (Table 4, Figure 2). For both compounds, a suppression of the parasite load was found (>99%) in both genders, and while 0% survival was noticed in either untreated male and female groups, CV-treated females and males resulted in 90 and 60% survival, respectively. DB1831 gave the most effective results, reaching 100 and 90% of animal survival, respectively, and rates of 2/10 and 7/10 free of post-immune suppression relapse for female and male mice, respectively (Table 4).

## 4. Discussion

*T. cruzi* can survive up to 250 days at room temperature and up to 18 days kept in whole blood stored at 4 °C [58]. Crystal violet was the first agent used for pathogen reduction [32], but due to side effects, its use was limited; thus, several studies were conducted in the search for improved antiparasitic compounds. 

To be considered an appropriate pathogen blood inactivating agent, some properties are required, including being active at the temperatures and pH at which blood components are stored, not cause damage to red cells, leukocytes, and platelets, and display a safe profile to the blood recipient. Quite a few compounds appear to fulfill such requirements. Some agents such as riboflavin and analogues, in combination with UV light exposure and leukoreduction, showed satisfactory results, but none fully inactivated the parasite in all essential blood preparations such as red blood cells, platelet concentrates and plasma components [37,59]. Thus, a novel and easy-to-use agent able to promote blood safety, not only against *T. cruzi*, but potentially against other blood-borne pathogens, would be highly welcome [60].

In the current study, the ability of three arylimidamides to reduce the viability of *T. cruzi* trypomastigote forms in experimentally infected mouse blood samples was assayed, aiming to identify novel chemotherapeutic or chemoprophylactic agents. These assays were first carried out in in vitro experiments in which bloodstream forms were spiked into whole blood samples artificially contaminated and treated with DB745, DB766 and DB1831. Our present findings demonstrated the rapid action of these arylimidamides on artificially contaminated whole blood samples exposed to blood bank component storage conditions (4 °C). This fast-killing profile (only 2 h of incubation) is an advantageous characteristic of some arylimidamides [45,47] since VG required an exposure time of 24 h for blood decontamination [60]. Similarly, in *Trypanosoma brucei*, short exposure times to some diamidines, including diminazene and DB75, from minutes to a few hours, resulted in the ultimate sterilization of parasite cultures in a dose-dependent way [61]. This was attributed to the observations that these compounds accumulate rapidly in the parasite [61], via a specific transporter identified as the purine carrier TbAT1 [62,63]. The implication that AIAs may be similarly taken up by a rapid transport process should be a priority in further studies of their utility against *T. cruzi*. 

Our in vitro and in vivo data demonstrate that at concentrations that are non-toxic to mouse blood samples (at their respective EC_50_ values and at 96 µM), *T. cruzi* invasion and intracellular proliferation was at least partially impaired. DB1831 was the most promising treatment at 96 µM, as in addition to suppressing the parasitemia by >99%, it gave protection against acute *T. cruzi* infection of both male and female mice, while all untreated animals died. As reported for sterol inhibitors and for Bz, male mice were more sensible to the DB1831-treated inoculum, as at 96 µM of the drug, 7 out of 10 mice remained free of parasitemia (as opposed to 2/10 female mice), even after immunosuppression. This confirms the relevance of using both genders during drug development analysis [64]. 

The implementation of universal diagnostic testing for CD should be mandatory in blood banks, as well as an increase in diagnosis and antiparasitic treatment of infected people; these are among the essential pillars for achieving the WHO plan of interrupting the four main CD transmission routes by 2030 (vectorial, blood transfusion, organ transplant and vertical transmission). In this sense, other strategies that may complement existing methods are relevant to further minimize the possible risk of infections via blood transfusion [59].

Our present findings demonstrate the prophylactic antiparasitic efficacy of arylimidamides, at concentrations that are non-toxic to red cells. The short duration (2 h) of the incubation employed here, resulting in the almost complete elimination of parasitemia, strongly suggests that full chemoprophylaxis of blood samples should be possible. We therefore argue that this class of compounds should be further evaluated regarding their potential use in blood banks for chemoprophylaxis, aiming to contribute to transmission control of CD by iatrogenic routes. *T. cruzi* is an emerging pathogen and considered to be an important public health concern, gaining special importance in historically nonendemic countries, where the major transmission routes are via blood transfusion and the congenital route [65]. Additional studies are also planned to improve parasite elimination using different approaches such as different AIAs dosages, drug combination and a longer period of blood exposure. 

Previous reports have demonstrated that the *T. cruzi* iatrogenic transmission varies according to many factors, including the amount of transfused blood elements, parasite strain, and presence of observable parasitemia at the time of donation, recipient immune status and screening tests, among others, as reviewed in [37]. In recent decades, to mitigate the risk of transfusion-transmitted Chagas, several strategies were implemented in different countries including risk factor assessments (through donor questionnaires and anamnesis) and specific serological testing, as well as alternative methods such as leukofiltration and pathogen inactivation [37]. 

However, these mitigation strategies have been unevenly implemented, varying from no control measures to more stringent approaches in both developing and developed countries [66]. Thus, (i) the occurrence of chronic asymptomatic and undiagnosed carriers with low and transient parasitemia in peripheral blood who are able to transmit the disease via blood transfusion, (ii) the use of variable control strategies according to each country (and sometimes region) depending on political will, resources and commitment, and (iii) the strong likelihood that the real number of transfusion cases may be underestimated and/or underreported [26,37], all support studies related to blood bank chemoprophylaxis for CD. 

## Figures and Tables

**Table 1:** Spectrophotometric analysis (540 nm) of the hemolytic action of arylimidamides on mouse blood samples.

	μM	OD	VI ^a^
Positive control ^b^	-	2.631 ± 0.096 ^d^	1.000 *
Negative control ^c^	-	0.212 ± 0.018	0.081
DB745	1.2	0.220 ± 0.063	0.084
	3.9	0.215 ± 0.037	0.082
	10.6	0.184 ± 0.014	0.070
	32.0	0.195 ± 0.007	0.074
	96	0.206 ± 0.004	0.078
DB766	1.2	nd ^e^	-
	3.9	0.248 ± 0.018	0.094
	10.6	0.200 ± 0.034	0.076
	32.0	0.230 ± 0.051	0.088
	96	0.317 ± 0.069	0.121 *

^a^ Variation index = OD(treated)/OD(control); ^b^ Incubation with H_2_O; ^c^ Incubation with PBS; ^d^ media ± standard deviation of one experiment performed in triplicate; ^e^ not determined; * *p* = 0.5606.

**Table 2:** *Trypanosoma cruzi* infectivity of cardiac cell cultures pretreated with DB745 and DB766 arylimidamides.

	Time (h)	% Infection	Par./Infected CC	II ^a^
Untreated	24	23.4 ± 5.5 ^b^	1.6 ± 0.5	38 ± 16
	72	26.3 ± 2.3	17.7 ± 1.7	466 ± 49
DB745 (6.4 μM)	24	7.3 ± 3.4 *	1.4 ± 0.3	10 ± 4.4
	72	14.6 ± 0.2	18.6 ± 2.4	272 ± 39
DB766 (1.14 μM)	24	1.0 ± 1.5 *	0.5 ± 0.7	0.5 ± 2.1
	72	0.1 ± 0.2	0.3 ± 0.4	0.03 ± 0.2

^a^ II (infection index) = % infection × parasites/infected CM; ^b^ media ± standard deviation of two experiments performed in duplicate. Statistical analysis of the % infection: untreated versus DB745-treated (24 h and 72 h: * *p* = 0.012 and *p* = 0.006, respectively) and untreated versus DB766-treated (24 h and 72 h: *p* = 0.012 and *p* = 0.0006, respectively. DB745 versus DB766 at 24 h and 72 h: *p* = 0.098 and *p* = 0.0001, respectively.

**Table 3:** In vivo infectivity of mice blood samples experimentally infected with *T. cruzi* bloodstream trypomastigotes previously treated with the AIAs and CV.

	Inoculation Pre-Treatment (μM)	Parasitemia at 8 dpi (×10^4^ Parasites/mL) and Clearance of the Parasitemia	
		# 1	# 2
Vehicle	-	261.1 ± 124.6	277.9 ± 57.2
		0/5	0/5
DB745	6.4 ^a^	156.4 ± 31.1	4.2 ± 2.1
		0/5	0/5
DB766	1.14 ^a^	141.3 ± 152.7	170.5 ± 39
		0/5	0/5
DB1831	0.5 ^a^	366.2 ± 295.1	157.7 ± 55.9
		0/5	0/5
DB745	96	4.6 ± 2.4	1.3 ± 1.1
		0/5	0/5
DB766	96	7.9 ± 5.2	0.7 ± 1.1
		0/5	0/5
DB1831	96	0.0 ± 0.0	0.0 ± 0.0
		2/5	4/5
CV	96	34.7 ± 38.5	1.6 ± 1.7
		0/5	0/5

^a^ EC_50_/2 h; Results of two independent experiments are listed, each n = 5 animals per group. Statistical analysis of the parasitemia peak level gave significancy (*p* < 0.05) in all groups treated with 96 μM concentrations versus the untreated group.

**Table 4:** The effect of the *T. cruzi* inocula previously treated by DB1831 and crystal violet at 96 µM during murine models (female and male Swiss mice) of acute experimental infection.

Treatment	Doses (μM)	Animal’s Gender	Parasitemia *	Clearance of the Parasitemia **
Vehicle	0	Female	247.7 ± 25.3	0/10
		Male	209.8 ± 29.2	0/10
DB1831	96	Female	0.1 ± 0.5	2/10
		Male	0.0 ± 0.0	7/10
CV	96	Female	0 ± 0	3/10
		Male	0.1 ± 0.2	4/10

n = 10 animals per group; * at 8 dpi (10^4^ parasites/mL); ** after cyclophosphamide administration.

## Data Availability

All relevant data are contained in the manuscript.
